# Gluten free diet is a cure not a poison! 

**Published:** 2015

**Authors:** Kamran Rostami, David Aldulaimi, Mohammad Rostami-Nejad

**Affiliations:** 1 *Gastroenterology Unit, Alexander Hospital Redditch, UK*; 2 *Gastroenterology and Liver Diseases Research Institute, Shahid Beheshti University of Medical Sciences, Tehran, Iran *

In the April issue, GHFBB is publishing a number of papers that focuses on the clinical and immunohistochemical diagnosis of gluten-related disorders. Dietary intervention for example, has revolutionised the treatment of patients with disabling irritable bowel syndrome (IBS). Increasingly, we are discovering that a large proportion of diagnoses such as IBS present with food-related bowel symptoms which mask the treatable presentation of either transient or permanent gluten sensitivity (1). A gluten-free diet is used by wide numbers of people and has been increasingly prescribed by health professionals over recent years for patients with “non-coeliac gluten-related” disorders. In particular, the use of a gluten free diet (GFD) has been proved to be very effective in a large proportion of patients labelled IBS. It has more recently become a very competitive therapeutic approach to other dietary interventions, like the removal of fermentable carbohydrates (FODMAP) (2). Improvement in the symptoms with a GFD, and their re-occurrence following the reintroduction of gluten seems to show that a large proportion of these patients, also fulfilling the Rome III criteria, have unrecognised gluten-sensitivity (3).

Intestinal symptoms with different aetiology (4) usually results in dysbiosis. Both fermentable carbohydrates such as those included in the term FODMAP, and a GFD, have some effect on intestinal microbiota (5, 6). FODMAP delivered to the colon have potential anti-carcinogenic and anti-inflammatory actions (7). The evidence shows that good pro-biotics, like *Bifidobacteria* spp, are reduced in those patients following a low FODMAP diet (8). Therefore, there is a need to be able to critically distinguish between gluten sensitivity and other carbohydrate sensitivities. Here, the start point in managing patients should be the Rome III criteria, since a reduction in FODMAPs delivered to colonic microbiota might have deleterious effects on the growth of bacteria which have potentially favourable health effects (9).

Some recent studies have raised the concerns that GFDs might be a risk factor for metabolic syndrome (10, 11). This might be explained by improvements in intestinal absorption as well as the high content of sugar, fat and calorie in gluten-free products: indeed, the high calorie content of gluten free products may seriously affect Leptin. Leptin resistance is considered a risk factor for obesity. It has been hypothesized that dietary cereal grain protein could increase circulating Leptin levels by preventing it from binding to Leptin receptors (12). But before jumping to conclusions, it shouldn’t be forgotten that the prevalence of those who are overweight or frankly obese has increased substantially worldwide in less than one generation (13) and that could include those with specific diseases including GRD (14). GFD is essential therapy in patients with GRD: Its benefits include inducing remission of inflammatory process, reducing its long-term complications, in addition to the important control and eradication of symptoms. Many patients consider GFD very balanced, healthy and useful for weight control due to its restrictive nature (unpublished data). Therefore, a GFD shouldn’t necessarily put these patients at higher risk for metabolic syndrome. Having said that, as a general rule, any treatment modality might potentially have undesirable effects and this information when available should be highlighted to potential candidates. The restriction of calorie intake should be applied as the standard strategy for patients considered high risk for metabolic syndrome and this intervention should include gluten and non-gluten containing nutrients. Finally, with respect to the histological analysis of intestinal mucosal specimens, we include a critical paper which articulates considerable doubt on the validity of Oberhuber’s subdivisions of the Marsh III lesion into a, b, c categories. These apparent distinctions have been widely, if not uncritically, employed but as this paper amply reveals, there are neither morphological nor quantitative bases to these subdivisions. From that, it follows that they play little role in the diagnosis, management, or longer-term treatment of patients with proven coeliac disease (15). In order to round off this special issue, we have included a histology consisting of diagrams and stained sections referable to GRDs.
